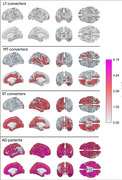# Local brain aging maps proximity to Alzheimer's‐related cognitive impairment

**DOI:** 10.1002/alz70856_107525

**Published:** 2026-01-09

**Authors:** Phoebe Imms, Nikhil N Chaudhari, Owen M Vega, Andrei Irimia

**Affiliations:** ^1^ Leonard Davis School of Gerontology, University of Southern California, Los Angeles, CA, USA; ^2^ Viterbi School of Engineering, University of Southern California, Los Angeles, CA, USA; ^3^ Dana and David Dornsife College of Arts & Sciences, University of Southern California, Los Angeles, CA, USA; ^4^ Kings College London, London, London, United Kingdom

## Abstract

**Background:**

Traditional brain morphometrics (e.g., regional brain volumes) may not be sensitive to early and subtle neurodegenerations that precede cognitive impairment (CI). Deep learning neural networks (DNNs) leverage neuroimaging data to detect voxel‐level deviations from normality. DNNs are trained to predict chronological age (CA) from magnetic resonance images (MRIs), resulting in a global brain age (BA) that estimates the biological age of a brain. The difference between BA and CA reflects the age gap (AG), which is larger in patients with neurodegenerative diseases. Our DNN generates *local* AGs in specific brain regions, to offer regionally interpretable insights into neurodegeneration.

**Method:**

We quantify global and local AGs in 1,320 participants across two large‐scale data repositories (i.e., NACC and ADNI). We compare group‐level differences in global AG to group‐level differences in brain volume across CN adults with and without CI in their future (converters and non‐converters, respectively), and individuals with Alzheimer's disease. Additionally, we explore regional‐level spatial changes in brain aging across CN adults who will convert in the short‐term (ST; 0.5 to 2.5 years), mid‐term (MT; 2.5 to 6 years), and long‐term (LT; > 6 years).

**Result:**

Global AGs, but not brain volumes, are significantly elevated in ST and MT converters and AD subjects compared to non‐converters. Compared to non‐converters, MT converters’ local AGs are significantly higher in temporal, insular, and orbitofrontal regions. As converters approach CI onset (i.e., ST converters), larger AGs propagate to anterior cingulate cortices, lateral temporal and frontal lobes, and parietal regions, before encompassing the rest of the cortex in subjects with AD.

**Conclusion:**

Our results challenge the assumption that *T*_1_‐weighted MRIs cannot reveal temporal dynamics of neurodegeneration. The patterns revealed by local AG maps recapitulate known neurodegenerative patterns of AD observed using imaging techniques that require exposure to radiation (i.e., positron emission tomography). The ability to employ spatiotemporally sensitive biomarkers that are non‐invasive and informative of proximity to CI conversion reduces barriers to early detection of Alzheimer's disease.